# Supplementary material for: Unidirectional chiral scattering from single enantiomeric plasmonic nanoparticles
Source: Nat Commun. 2025 Jan 28;16:1125. doi: 10.1038/s41467-024-55277-9 (PMC11775228; doi:10.1038/s41467-024-55277-9)
Supplement: Supplementary file 1 — Supplementary Information [file 41467_2024_55277_MOESM1_ESM.pdf]

Supplementary Information
Unidirectional chiral scattering from single enantiomeric plasmonic nanoparticles

Yuanyang Xie*,#, Alexey V. Krasavin#, Diane J. Roth, and Anatoly V. Zayats*

Department of Physics and London Centre for Nanotechnology,

King's College London, London, WS2R 2LS, UK

**Email Address: yuanyang.xie@kcl.ac.uk, a.zayats@kcl.ac.uk,*

# These authors contributed equally to this work.

Supplementary Note 1. Complete basis formed by rotating chiral dipoles.

It is straightforward to explicitly express chiral dipoles rotating around the z-axis in terms of electric and magnetic dipoles in the xy-plane:

$$\begin{pmatrix} |\boldsymbol{\sigma}_z^{++}\rangle \\ |\boldsymbol{\sigma}_z^{+-}\rangle \\ |\boldsymbol{\sigma}_z^{-+}\rangle \\ |\boldsymbol{\sigma}_z^{--}\rangle \end{pmatrix} = \begin{pmatrix} \mathrm{i} & -1 & 1 & \mathrm{i} \\ \mathrm{i} & 1 & 1 & -\mathrm{i} \\ -\mathrm{i} & 1 & 1 & \mathrm{i} \\ -\mathrm{i} & -1 & 1 & -\mathrm{i} \end{pmatrix} \begin{pmatrix} |\mathbf{p}_x\rangle \\ |\mathbf{p}_y\rangle \\ |\mathbf{m}_x\rangle \\ |\mathbf{m}_y\rangle \end{pmatrix}, \tag{1}$$

where the value of a magnetic dipole component was divided by c to match the norm of $|\mathbf{m}\rangle$ in the chiral dipole definition. Inverting the matrix, one can obtain how electric and magnetic dipolar states in the xy-plane can be expressed in terms of chiral dipoles rotating around the z-axis:

$$\begin{pmatrix} |\mathbf{p}_x\rangle \\ |\mathbf{p}_y\rangle \\ |\mathbf{m}_x\rangle \\ |\mathbf{m}_y\rangle \end{pmatrix} = \frac{1}{4} \begin{pmatrix} -\mathrm{i} & -\mathrm{i} & \mathrm{i} & \mathrm{i} \\ -1 & 1 & 1 & -1 \\ 1 & 1 & 1 & 1 \\ -\mathrm{i} & \mathrm{i} & -\mathrm{i} & \mathrm{i} \end{pmatrix} \begin{pmatrix} |\boldsymbol{\sigma}_z^{++}\rangle \\ |\boldsymbol{\sigma}_z^{+-}\rangle \\ |\boldsymbol{\sigma}_z^{-+}\rangle \\ |\boldsymbol{\sigma}_z^{--}\rangle \end{pmatrix}. \tag{2}$$

From this matrix equation one can derive that electric dipoles rotating in the xy-plane clockwise and counterclockwise (looking along the z-axis) are represented by $-\mathrm{i}/2 \cdot (|\boldsymbol{\sigma}_z^{++}\rangle - |\boldsymbol{\sigma}_z^{-+}\rangle)$ and $\mathrm{i}/2 \cdot (|\boldsymbol{\sigma}_z^{--}\rangle - |\boldsymbol{\sigma}_z^{+-}\rangle)$, respectively, by combining chiral dipoles with the opposite handedness rotating in the same direction (Supplementary Fig. 1a). For rotating magnetic dipoles the corresponding expressions are $1/2 \cdot (|\boldsymbol{\sigma}_z^{++}\rangle + |\boldsymbol{\sigma}_z^{-+}\rangle)$ and $1/2 \cdot (|\boldsymbol{\sigma}_z^{--}\rangle + |\boldsymbol{\sigma}_z^{+-}\rangle)$, respectively. Similarly, two Huygens dipoles emitting in the opposite directions along the z-axis $|\mathbf{p}_x\rangle + |\mathbf{m}_y\rangle$ and $|\mathbf{p}_y\rangle + |\mathbf{m}_x\rangle$ are represented by $-\mathrm{i}/2 \cdot (|\boldsymbol{\sigma}_z^{++}\rangle - |\boldsymbol{\sigma}_z^{--}\rangle)$ and $1/2 \cdot (|\boldsymbol{\sigma}_z^{+-}\rangle + |\boldsymbol{\sigma}_z^{-+}\rangle)$, respectively (Supplementary Fig. 1b). For Janus dipoles, combinations of all four rotating chiral dipoles are needed.

Overall, it can be seen that any combination of $|\mathbf{p}_x\rangle$, $|\mathbf{p}_y\rangle$, $|\mathbf{m}_x\rangle$ and $|\mathbf{m}_y\rangle$, i.e., an arbitrary dipolar excitation within the same plane (which is frequently the case due to the illumination conditions and the symmetry of the scatterer [1]) can be represented by a combination of four rotating dipoles. A complete set for the description of any dipolar excitation in three dimensions is produced by six rotating chiral dipoles (four will describe the rotation in the

xy-plane and four more are needed to compose the remaining $|\mathbf{p}_z\rangle$ and $|\mathbf{m}_z\rangle$ components, only two of the latter set being independent).

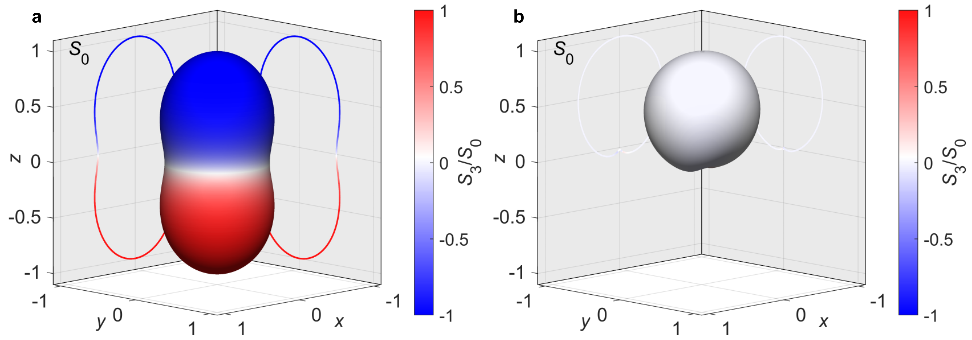

Supplementary Fig. 1. **Far-field radiation intensity/polarisation diagrams of a circularly polarised (rotating) electric dipole and a Huygens dipole.** **a** A circularly polarised electric dipole $(\mathrm{i}/2 \cdot |\boldsymbol{\sigma}_z^{--}\rangle - |\boldsymbol{\sigma}_z^{+-}\rangle)$. **b** A Huygens dipole $(-\mathrm{i}/2 \cdot (|\boldsymbol{\sigma}_z^{++}\rangle - |\boldsymbol{\sigma}_z^{--}\rangle)$. The colour map shows S_3/S_0 Stokes parameter which represents the handedness of the emitted light (values 1 and -1 correspond RCP and LCP, respectively, defined from the point of view of the source).

Supplementary Note 2. Multipole decomposition.

The implemented multipole decomposition method is based on the analysis of the numerically calculated polarisation current $\mathbf{J} = -\mathrm{i}\omega\epsilon_0[\epsilon_r - \epsilon_{r,d}]\mathbf{E}$ [2]. The multipole contributions were calculated as [3]

$$
\begin{aligned}
p_\alpha =& -\frac{1}{\mathrm{i}\omega}\Big\{\int \mathrm{d}^3\mathbf{r} J_\alpha \mathrm{j}_0(kr) \\
& + \frac{k^2}{2}\int \mathrm{d}^3\mathbf{r}\Big[3(\mathbf{r}\cdot\mathbf{J})r_\alpha - r^2 J_\alpha\Big]\frac{\mathrm{j}_2(kr)}{(kr)^2}\Big\}, \\
m_\alpha =& \frac{3}{2}\int \mathrm{d}^3\mathbf{r}(\mathbf{r}\times\mathbf{J})_\alpha \frac{\mathrm{j}_1(kr)}{(kr)^2}, \\
Q^e_{\alpha\beta} =& -\frac{3}{\mathrm{i}\omega}\Big\{\int \mathrm{d}^3\mathbf{r}\Big[3(r_\beta J_\alpha + r_\alpha J_\beta) - 2(\mathbf{r}\cdot\mathbf{J})\delta_{\alpha\beta}\Big]\frac{\mathrm{j}_1(kr)}{kr} \\
& + 2k^2\int \mathrm{d}^3\mathbf{r}\Big[5r_\alpha r_\beta(\mathbf{r}\cdot\mathbf{J}) - (r_\alpha J_\beta + r_\beta J_\alpha)r^2 \\
& - r^2(\mathbf{r}\cdot\mathbf{J})\delta_{\alpha\beta}\Big]\frac{\mathrm{j}_3(kr)}{(kr)^3}\Big\}, \\
Q^m_{\alpha\beta} =& 15\int \mathrm{d}^3\mathbf{r}\Big\{r_\alpha(\mathbf{r}\cdot\mathbf{J})_\beta + r_\beta(\mathbf{r}\cdot\mathbf{J})_\alpha\Big\}\frac{\mathrm{j}_2(kr)}{(kr)^2},
\end{aligned}
\tag{3}
$$

where p_α, m_α, $Q^e_{\alpha\beta}$ and $Q^m_{\alpha\beta}$ ($\alpha,\beta = x,y,z$) are the electric dipole, magnetic dipole, electric quadrupole and magnetic quadrupole moments, respectively, $\mathbf{J}$ is the induced polarisation current, $\mathrm{j}_n(kr)$ is the spherical Bessel functions with k and r being the wavenumber and the distance to the origin, respectively (note an incorrect sign in the expression for the polarisation current in the source reference [2, 4] which was corrected here).

The contributions of the multipoles to the total scattering intensity are given by [3]

$$
\begin{aligned}
C^{\mathrm{tot}}_{\mathrm{scat}} =& \frac{k^4}{6\pi\epsilon_0^2|\mathbf{E}_{\mathrm{inc}}|^2}\Big[\sum_\alpha\Big(|p_\alpha|^2 + \Big|\frac{m_\alpha}{\mathrm{c}}\Big|^2\Big) + \\
& \frac{1}{120}\sum_{\alpha\beta}\Big(|kQ^e_{\alpha\beta}|^2 + \Big|\frac{kQ^m_{\alpha\beta}}{\mathrm{c}}\Big|^2\Big) + \cdots\Big],
\end{aligned}
\tag{4}
$$

where $C^{\mathrm{tot}}_{\mathrm{scat}}$ is the total scattering cross-section and $|\mathbf{E}_{\mathrm{inc}}|$ is the electric field amplitude of the incident plane wave.

The decomposition into into chiral dipoles can be derived as

$$
\begin{aligned}
\sigma_\alpha^+ =& \frac{1}{2\omega}\Big\{\int \mathrm{d}^3\mathbf{r} J_\alpha \mathrm{j}_0(kr) \\
&+ \frac{3}{2\mathrm{c}\omega}\int \mathrm{d}^3\mathbf{r}(\mathbf{r}\times\mathbf{J})_\alpha \frac{\mathrm{j}_1(kr)}{(kr)^2} \\
&+ \frac{k^2}{2}\int \mathrm{d}^3\mathbf{r}\Big[3(\mathbf{r}\cdot\mathbf{J})r_\alpha - r^2 J_\alpha\Big]\frac{\mathrm{j}_2(kr)}{(kr)^2}\Big\}, \\
\sigma_\alpha^- =& -\frac{1}{2\omega}\Big\{\int \mathrm{d}^3\mathbf{r} J_\alpha \mathrm{j}_0(kr) \\
&- \frac{3}{2\mathrm{c}\omega}\int \mathrm{d}^3\mathbf{r}(\mathbf{r}\times\mathbf{J})_\alpha \frac{\mathrm{j}_1(kr)}{(kr)^2} \\
&+ \frac{k^2}{2}\int \mathrm{d}^3\mathbf{r}\Big[3(\mathbf{r}\cdot\mathbf{J})r_\alpha - r^2 J_\alpha\Big]\frac{\mathrm{j}_2(kr)}{(kr)^2}\Big\},
\end{aligned} \tag{5}
$$

where σ_α^+ and σ_α^- ($\alpha = x, y, z$) are the magnitudes for right-handed chiral dipole and left-handed chiral dipole, respectively. Their contributions to the scattering intensity is

$$
C_{\mathrm{scat}}^{\mathrm{dipole}} = \frac{k^4}{3\pi\epsilon_0^2|\mathbf{E}_{\mathrm{inc}}|^2}\Big[\sum_\alpha\Big(|\sigma_\alpha^+|^2 + |\sigma_\alpha^-|^2\Big)\Big]. \tag{6}
$$

Supplementary Note 3. Conversion between electric/magnetic and chiral dipolar bases.

To derive the conversion between electric/magnetic and chiral dipolar bases, as an example, the x-axis component of an arbitrary dipolar state is expressed both in the chiral and the electric/magnetic dipolar bases:

$$
\begin{aligned}
&\sigma_x^+(1,0,0)\left|\boldsymbol{\sigma}^+\right\rangle + \sigma_x^-(1,0,0)\left|\boldsymbol{\sigma}^-\right\rangle = \\
&= \sigma_x^+(1,0,0)[|\mathbf{m}\rangle + \mathrm{i}\,|\mathbf{p}\rangle] + \sigma_x^-(1,0,0)[|\mathbf{m}\rangle - \mathrm{i}\,|\mathbf{p}\rangle] = \\
&= m_x/\mathrm{c}(1,0,0)\,|\mathbf{m}\rangle + p_x(1,0,0)\,|\mathbf{p}\rangle\,.
\end{aligned} \tag{7}
$$

where the value of a magnetic dipole component was divided by c to match the norm of $|\mathbf{m}\rangle$ in the chiral dipole definition. Equalizing the terms in front of $|\mathbf{m}\rangle$, one obtains

$$
\begin{aligned}
\sigma_x^+ + \sigma_x^- &= m_x/\mathrm{c}, \\
\mathrm{i}\sigma_x^+ - \mathrm{i}\sigma_x^- &= p_x,
\end{aligned} \tag{8}
$$

and

$$\sigma_x^+ = \frac{m_x/\mathrm{c} - \mathrm{i}p_x}{2},$$
$$\sigma_x^- = \frac{m_x/\mathrm{c} + \mathrm{i}p_x}{2}. \tag{9}$$

Performing the same procedure for the other two components, one arrives to

$$\boldsymbol{\sigma}^+ = \frac{\mathbf{m}/\mathrm{c} - \mathrm{i}\mathbf{p}}{2},$$
$$\boldsymbol{\sigma}^- = \frac{\mathbf{m}/\mathrm{c} + \mathrm{i}\mathbf{p}}{2}, \tag{10}$$

used in the main text.

Supplementary Note 4. Derivation of the excitation of rotating chiral dipole by circularly polarised electromagnetic wave.

As shown in the main text, under the resonant conditions, the main dipolar modes excited in a nanohelicoid by an x-polarised plane electromagnetic wave are the electric dipole along x-direction $(p_x, 0, 0)\,|\mathbf{p}\rangle$ and, out of phase to it, a magnetic dipole $(0, -m_y, 0)\,|\mathbf{m}\rangle$, with the real-valued magnitudes related to each other as $p_x = m_y/\mathrm{c}$. Similarly, a y-polarised plane electromagnetic wave excites $(0, p_y, 0)\,|\mathbf{p}\rangle$ and $(m_x, 0, 0)\,|\mathbf{m}\rangle$ (note the change of sign for m due to rotation of the excitation and the excited dipoles by $\pi/2$. In the chosen simulation framework, the electric field of the incident circularly polarised wave propagating along the z-axis is given by $\mathbf{E}^{\mp} = (1, \pm\mathrm{i}, 0)E_0$, where $\mathbf{E}^+$ and $\mathbf{E}^-$ correspond to the RCP and LCP fields, respectively. Therefore, the major excited dipolar modes are (top sign for RCP, bottom for LCP)

$$\begin{aligned}
&(p_x, 0, 0)\,|\mathbf{p}\rangle + (0, -m_y, 0)\,|\mathbf{m}\rangle \pm \mathrm{i}[(0, p_y, 0)\,|\mathbf{p}\rangle + (m_x, 0, 0)\,|\mathbf{m}\rangle)] = \\
&= \pm\mathrm{i}(m_x, 0, 0,)\,|\mathbf{m}\rangle + (p_x, 0, 0)\,|\mathbf{p}\rangle - (0, m_y, 0)\,|\mathbf{m}\rangle \pm \mathrm{i}(0, p_y, 0)\,|\mathbf{p}\rangle = \\
&= \pm\mathrm{i}\{(m_x, 0, 0)\,|\mathbf{m}\rangle \mp \mathrm{i}(p_x, 0, 0)\,|\mathbf{p}\rangle \pm \mathrm{i}(0, m_y, 0)\,|\mathbf{m}\rangle + (0, p_y, 0)\,|\mathbf{p}\rangle\} = \\
&= \pm\mathrm{i}\{(m_x, 0, 0)\,|\mathbf{m}\rangle \mp \mathrm{i}(p_y, 0, 0)\,|\mathbf{p}\rangle \pm \mathrm{i}[(0, m_y, 0)\,|\mathbf{m}\rangle \mp \mathrm{i}(0, p_y, 0)\,|\mathbf{p}\rangle]\} = \\
&= \pm\mathrm{i}\{(\sigma, 0, 0)\,\left|\boldsymbol{\sigma}^{\mp}\right\rangle \pm \mathrm{i}(0, \sigma, 0)\,\left|\boldsymbol{\sigma}^{\mp}\right\rangle\} = \pm\mathrm{i}(\sigma, \pm\mathrm{i}\sigma, 0)\,\left|\boldsymbol{\sigma}^{\mp}\right\rangle = \pm\mathrm{i}\,\left|\boldsymbol{\sigma}_z^{\mp\pm}\right\rangle,
\end{aligned} \tag{11}$$

where it is taken into account that $\sigma_x = \sigma_y = \sigma$ due to the initial relations between p_x and m_y (p_y and m_x). Therefore, in the numerical simulations, circularly polarised LCP

and RCP light, propagating along the z-axis, excites $|\boldsymbol{\sigma}_z^{-+}\rangle$ and $|\boldsymbol{\sigma}_z^{+-}\rangle$ modes, respectively, corresponding to the far-field intensity and polarisation patterns presented in Fig. 1c,f in the main text.

Supplementary Note 5. Scattering from nanospheres and nanocubes.

For comparison, 180 nm diameter gold nanospheres and 180 nm gold nanocubes were simulated under circularly (LCP) polarised illumination (Supplementary Figs. 2 and 3). In both cases, electric dipoles along x- and y- directions are the dominant terms in the multipole decomposition with a substantial presence of electric quadrupole modes and a minor presence of magnetic dipoles (Supplementary Figs. 2a and 3a). In the case of the nanocubes, which are close in the geometry to the nanohelicoids, the amplitude of the magnetic dipole is somewhat larger. As a result of the interference of electric dipole and electric quadrupole modes, asymmetry between forward and backward scattering is observed [5, 6] (Supplementary Figs. 2b and 3b), which is particularly pronounced in the nanocubes, but still far from the case of the excitation of pure rotating chiral dipoles in the nanohelicoids. This asymmetry is clear in the difference in the integrated intensities of the RCP and LCP scattered light (Supplementary Figs. 2c and 3c). Here, the difference is also smaller in the nanosphere case, for which there is a crossing point marking equal LCP and RCP signals (Fig. 2c), which corresponds to equal magnitudes of the excited $|\boldsymbol{\sigma}^-\rangle$ and $|\boldsymbol{\sigma}^+\rangle$ chiral dipoles (Fig. 2d), or, taking into account a $-\pi/2$ phase difference between their x- and y- components, $|\boldsymbol{\sigma}_z^{-+}\rangle$ and $|\boldsymbol{\sigma}_z^{++}\rangle$ in the rotating chiral dipole basis. Both for the nanosphere and nanocube cases, in the chiral decomposition of the dipolar scattering contributions, $|\boldsymbol{\sigma}^-\rangle$ or $|\boldsymbol{\sigma}^+\rangle$ chiral dipoles are excited along x- and y- directions with equal magnitudes and a $-\pi/2$ phase difference defined by the LCP illumination (Figs. 2d and 3d). The asymmetry between σ^+ and σ^- magnitudes is related to the excitation of the magnetic dipoles, and is more pronounced in the nanocube case. The achiral nature of the objects is reflected in an equivalence of their interaction with LCP and RCP light, when all the related handedness characteristics are just changed to the opposite.

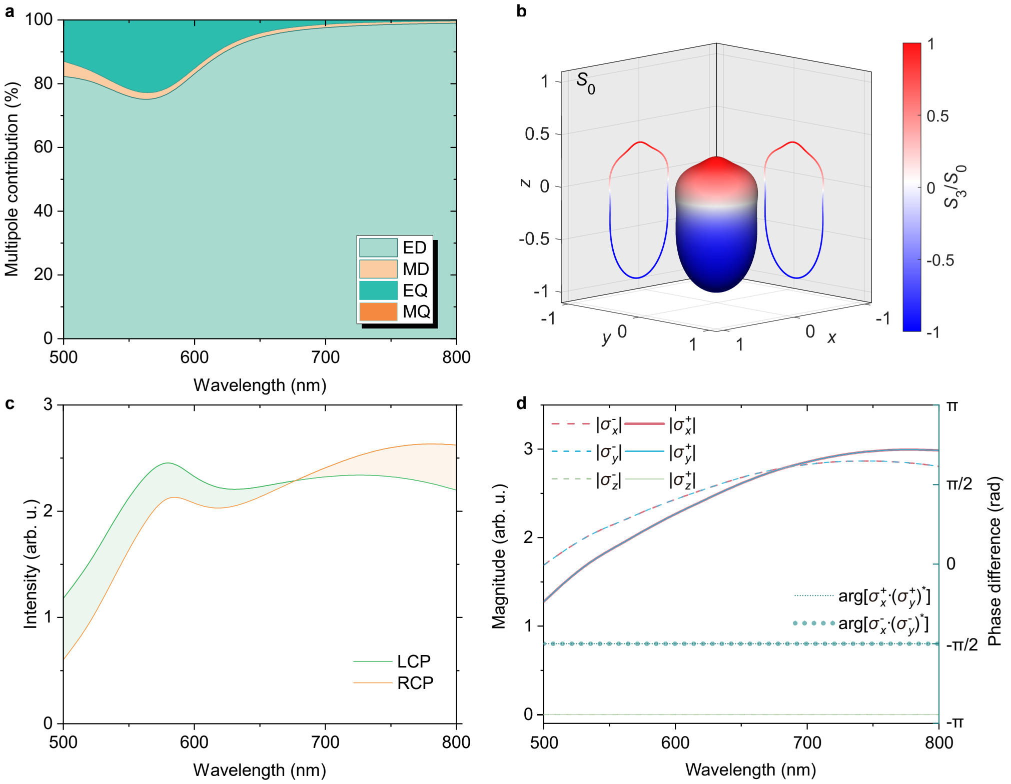

Supplementary Fig. 2. **Numerically simulated scattering from a gold nanosphere under LCP illumination.** **a** The multipole scattering efficiency ratios of a LCP light by a gold nanosphere with a 180 nm diameter in a SiO_2 matrix. **b** Far-field scattering intensity diagrams under 620 nm illumination (for comparison to the nanohelicoid case). The colour indicates the polarisation state of the scattered light calculated from Stokes parameters S_3/S_0 (1: RCP; -1: LCP). **c** Integrated intensities of RCP (orange) and LCP (green) scattered light. Colour of the shading indicate the higher intensity of the respective handedness. **d** The corresponding magnitudes σ^+ (solid line) and σ^- (dashed line) of the excited chiral dipoles, together with a phase difference between them (dark green dot line). Source data are provided as a Source Data file.

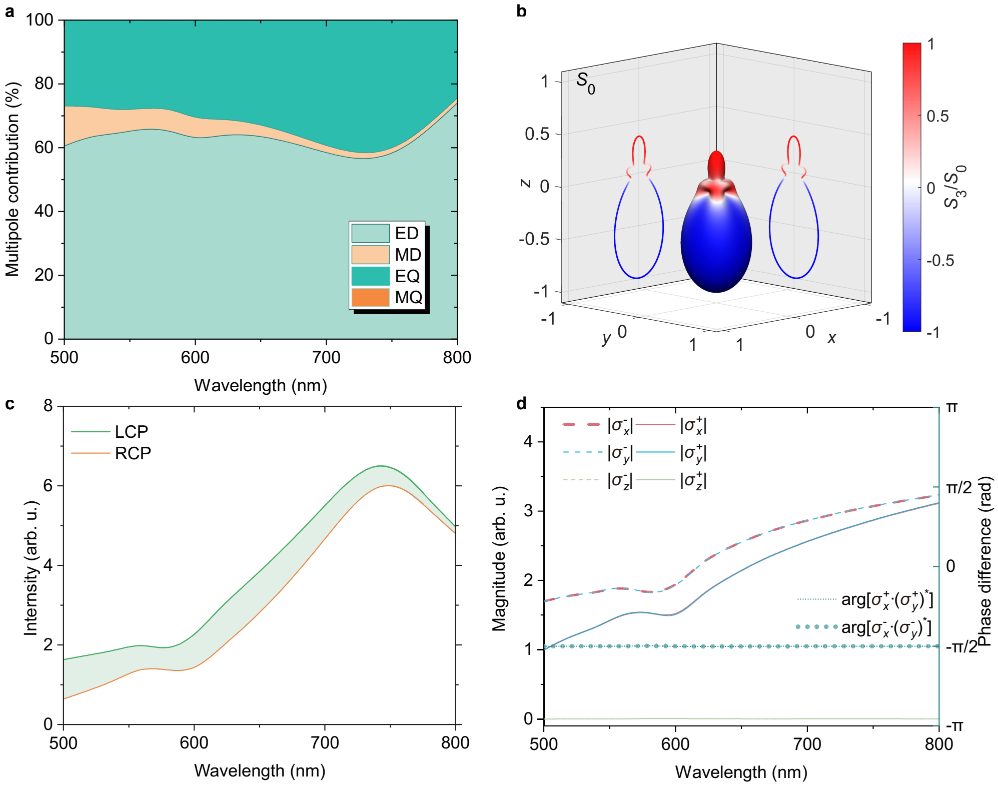

Supplementary Fig. 3. **Numerically simulated scattering from a gold nanocube under LCP illumination.** **a** The multipole scattering efficiency ratios of a LCP light by a 180 nm gold nanocube in a SiO_2 matrix, the nanocube edges and corners were rounded with a 4.5 nm radius to avoid field singularities. **b** Far-field scattering intensity and polarisation diagrams under 620 nm illumination (for comparison to the nanohelicoid case). The colour indicates the polarisation state of the scattered light calculated from Stokes parameters S_3/S_0 (1: RCP; -1: LCP). **c** Integrated intensities of RCP (orange) and LCP (green) scattered light. Colour of the shading indicate the higher intensity of the respective handedness. **d** The corresponding magnitudes σ^+ (solid line) and σ^- (dashed line) of the excited chiral dipoles, together with a phase difference between them (dark green dot line). Source data are provided as a Source Data file.

Supplementary Note 6. Scattering on differently oriented nanohelicoids.

In the experimental measurements, there maybe variations in the orientation of the nanohelicoids on the substrate with respect to the plane of incidence. To understand its importance, numerical simulations of scattering on a nanohelicoid rotated with respect to the incident light direction have been performed for three major nanohelicoid orientations: with light incident on the face, edge and corner (Supplementary Fig. 4). Both the directionality of the scattering and its chiral nature (the difference in the scattering intensity for LCP and RCP illumination) are not affected by the nanohelicoid orientation. This should be expected as the excitation of the multipoles responsible for chiral scattering does not depend on the nanoparticle orientation. The small differences in the backward scattering maybe due to the phase difference between the x- and y- dipolar components due to the orientation resulting in an "elliptical" chiral dipole and the increased contribution of the electric quadrupole excitations in the rotated nanohelicoids.

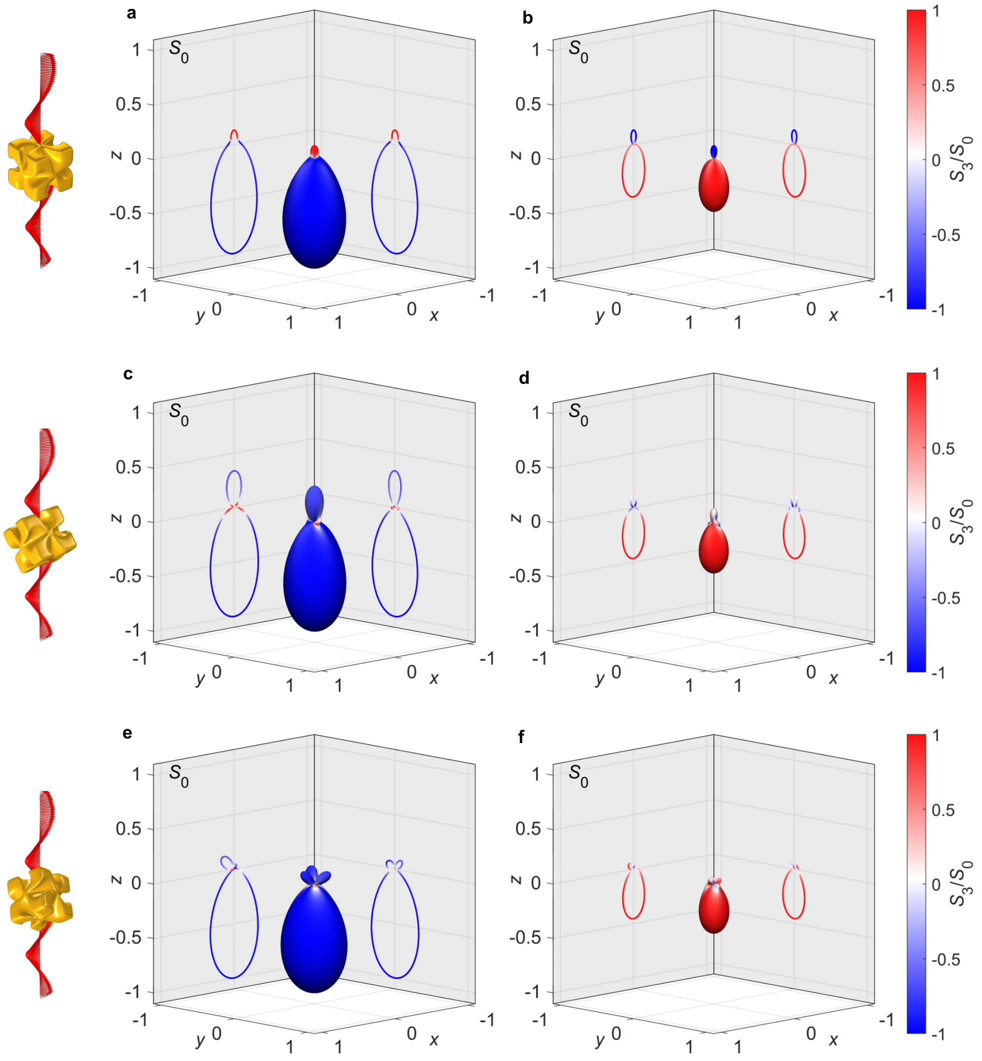

Supplementary Fig. 4. **Scattering on L-nanohelicoids with various orientations.** Far-field scattering intensity and polarisation diagrams under 620-nm-wavelength illumination incident on (**a**, **b**) face, (**c**, **d**) edge and (**e**, **f**) corner of a 180 nm nanohelicoid for (**a**, **c**, **e**) LCP and (**b**, **d**, **f**) RCP illumination.

Supplementary Note 7. Enantio-sensitive optical force on nanohelicoid due to directional scattering.

Directional scattering due to the excitation of the rotating chiral dipoles, controlled by the chirality of the illuminating light, also leads to different optical forces acting on a nanohelicoid under LCP and RCP illumination. The time-averaged optical force exerted on a particle has been calculated by integrating the Maxwell's stress tensor over any closed surface surrounding the nanoparticle S [7]:

$$\mathbf{F} = \oiint_S \overleftrightarrow{\mathbf{T}} \cdot \mathbf{n}\, \mathrm{d}s, \tag{12}$$

where $\mathbf{n}$ is the outward unit vector perpendicular to the integral area ds and $\overleftrightarrow{\mathbf{T}}$ is the Maxwell's stress tensor in a medium:

$$T_{ij} \equiv \epsilon_{\mathrm{r}}\epsilon_0(E_iE_j - \frac{1}{2}\delta_{ij}E^2) + \frac{1}{\mu_{\mathrm{r}}\mu_0}(B_iB_j - \frac{1}{2}\delta_{ij}B^2)). \tag{13}$$

Here, $\mathbf{E}$ and $\mathbf{B}$ are the electric and magnetic fields, respectively. The indices i and j refer to the coordinates x, y and z. The Kronecker delta δ_{ij} is equal to 1 if the indices are identical and zero otherwise. The ϵ_r and ϵ_0 (μ_r and μ_0) are the relative permittivities (permeabilities) of the surrounding medium and vacuum, respectively. A spherical surface S around the nanohelicoid was used for the integration [8, 9]. The results for the calculated z-component of the optical force (x- and y- components are zero due to the symmetry) for four combinations of the enantiomeric state of the nanohelicoid and the handedness of the incident circularly polarised light are presented in Supplementary Fig. 5. The largest difference between the force acting on a given nanohelicoid enantiomer for LCP and RCP is observed close to the wavelength of a rotating chiral dipole resonance at 620 nm. The relative difference expressed by the force g-factor

$$g_{\mathrm{force}} = 2\frac{F^{\mathrm{LCP}}_{\mathrm{optical}} - F^{\mathrm{RCP}}_{\mathrm{optical}}}{F^{\mathrm{LCP}}_{\mathrm{optical}} + F^{\mathrm{RCP}}_{\mathrm{optical}}} \tag{14}$$

is symmetric with opposite values for the different handedness of light and reaches large values ≈ 0.4. This effect can be used for chiral-sensitive nanoparticle sorting.

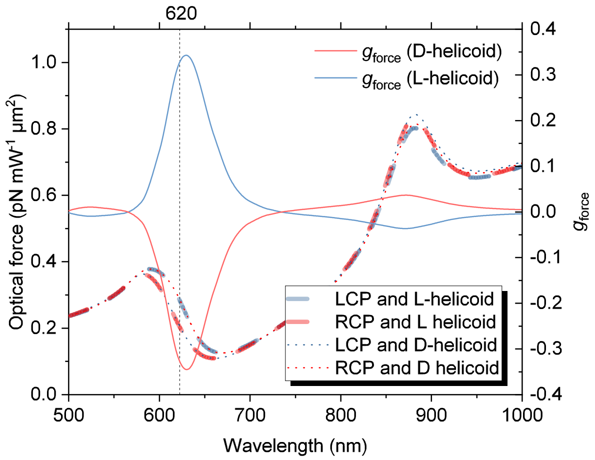

Supplementary Fig. 5. **Enantio-sensitive optical forces acting on nanohelicoids.** The optical force on 180 nm helicoids with (dashed line) L- and (dot line) D-chirality under (blue) LCP or (red) RCP illumination. The relative force difference for LCP and RCP illuminations on (blue solid line) L-helicoid and (red solid line) D-helicoids. Source data are provided as a Source Data file.

Supplementary Figures

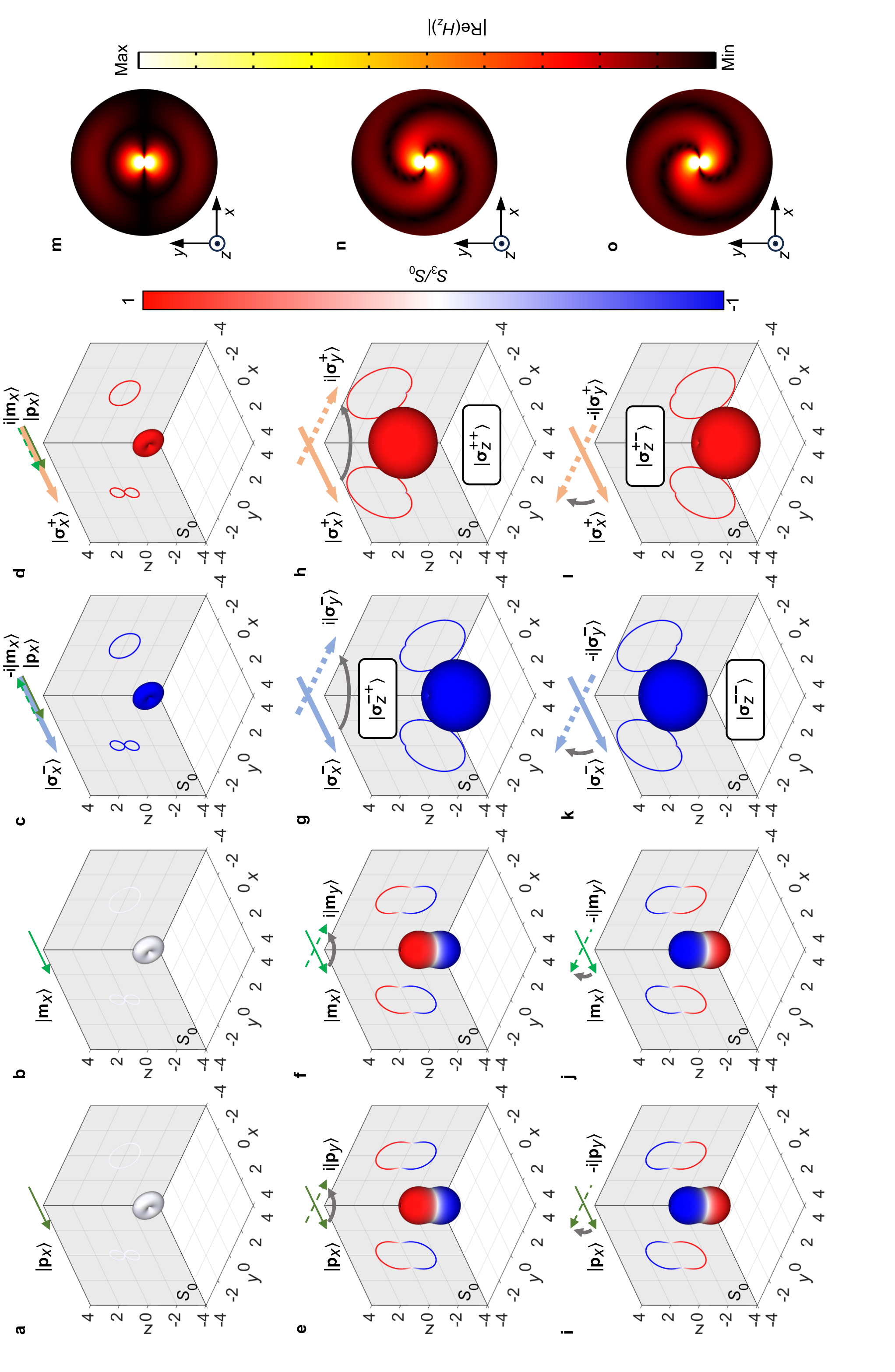

Supplementary Fig. 6. **Comparison of electric, magnetic and chiral dipoles, as well as their rotating variants.** **a–l** Schematics of (**a**) electric, (**b**) magnetic and (**c**,**d**) chiral dipoles with (**c**) left and (**d**) right handedness, together with their variants rotating (**e–h**) clockwise and (**i–l**) counterclockwise, accompanied by the corresponding far-field radiation intensity/polarisation diagrams (intensities in all diagrams were identically normalised, in such a way that the maximum intensity value for the non-rotating dipoles is 1. **m–o** Near-field radiation patterns (absolute value of the real part of the z-component of the magnetic field H_z plotted in the xy-plane), identical for all dipolar states in each row.

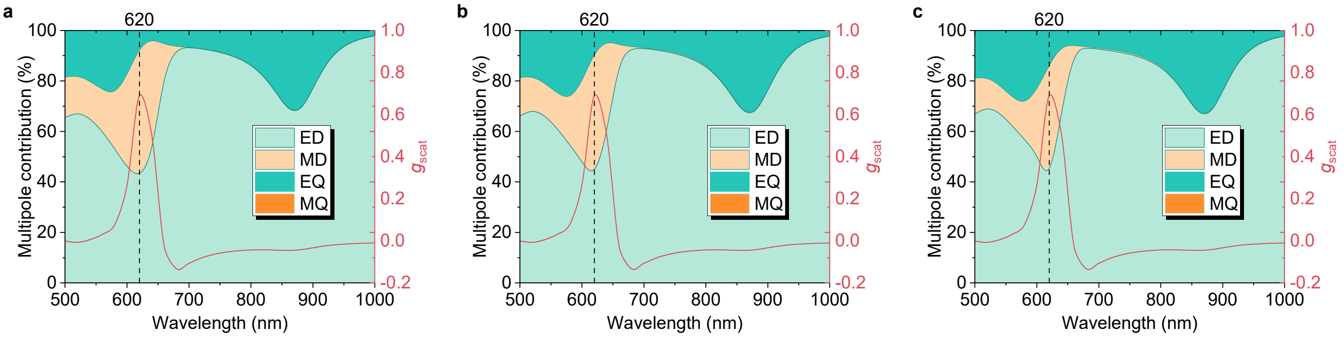

Supplementary Fig. 7. **Multipolar response of a plasmonic L-nanohelicoid. a–c** Multipolar contributions to the scattering intensity for (**a**) LCP, (**b**) linearly polarized and (**c**) RCP illumination calculated with the scattering cross-section formalism presented in Supplementary Note 2 together with the corresponding scattering g-factor (identical for both plots). Source data are provided as a Source Data file.

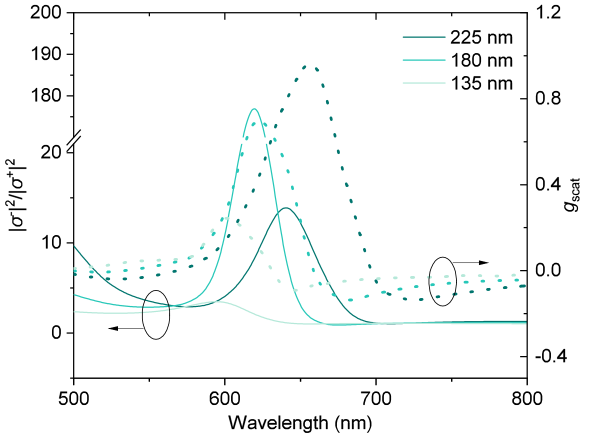

Supplementary Fig. 8. **Chiral response of plasmonic L-nanohelicoids with various sizes.** The ratio of squares of the amplitudes of right- and left-handed chiral dipoles $\boldsymbol{\sigma}^{\pm} = (\mathbf{m}/\mathrm{c} \mp i\mathbf{p})/2$ obtained from the numerical simulations using Suppl. Eqs. (1) and (2) (solid lines) and the corresponding scattering g-factor (dashed lines) for L-nanohelicoids of various sizes in a SiO_2 matrix under RCP illumination. Source data are provided as a Source Data file.

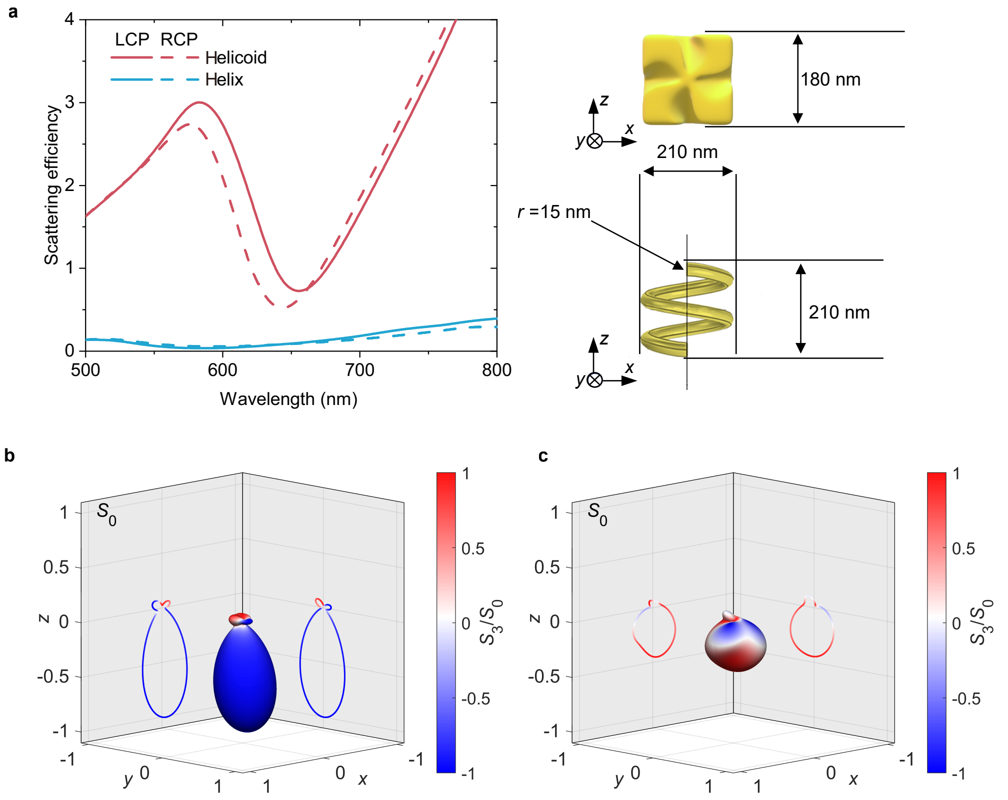

Supplementary Fig. 9. **Comparison of scattering on nanohelicoid and nanohelix.** (**a**) The simulated scattering efficiency for left-handed gold helix and helicoid nanoparticles of comparable sizes in SiO_2. (**b, c**) Far-field scattering intensity and polarisation diagrams of the right-handed helix under 620 nm (b) LCP and (c) RCP illumination. Source data are provided as a Source Data file.

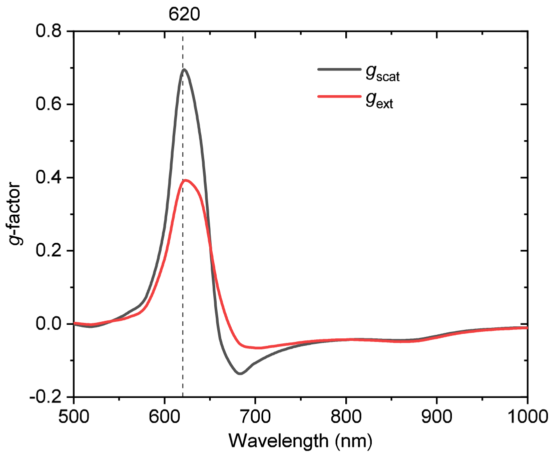

Supplementary Fig. 10. **Numerically simulated scattering and extinction *g*-factors of L-nanohelicoid.** The g-factors were calculated using (red) the extinction cross-section $(2(C_{\text{ext}}^{\text{LCP}} - C_{\text{ext}}^{\text{RCP}})/(C_{\text{ext}}^{\text{LCP}} + C_{\text{ext}}^{\text{RCP}}))$ and (black) the scattering cross-section $(2(C_{\text{scat}}^{\text{LCP}} - C_{\text{scat}}^{\text{RCP}})/(C_{\text{scat}}^{\text{LCP}} + C_{\text{scat}}^{\text{RCP}}))$. Source data are provided as a Source Data file.

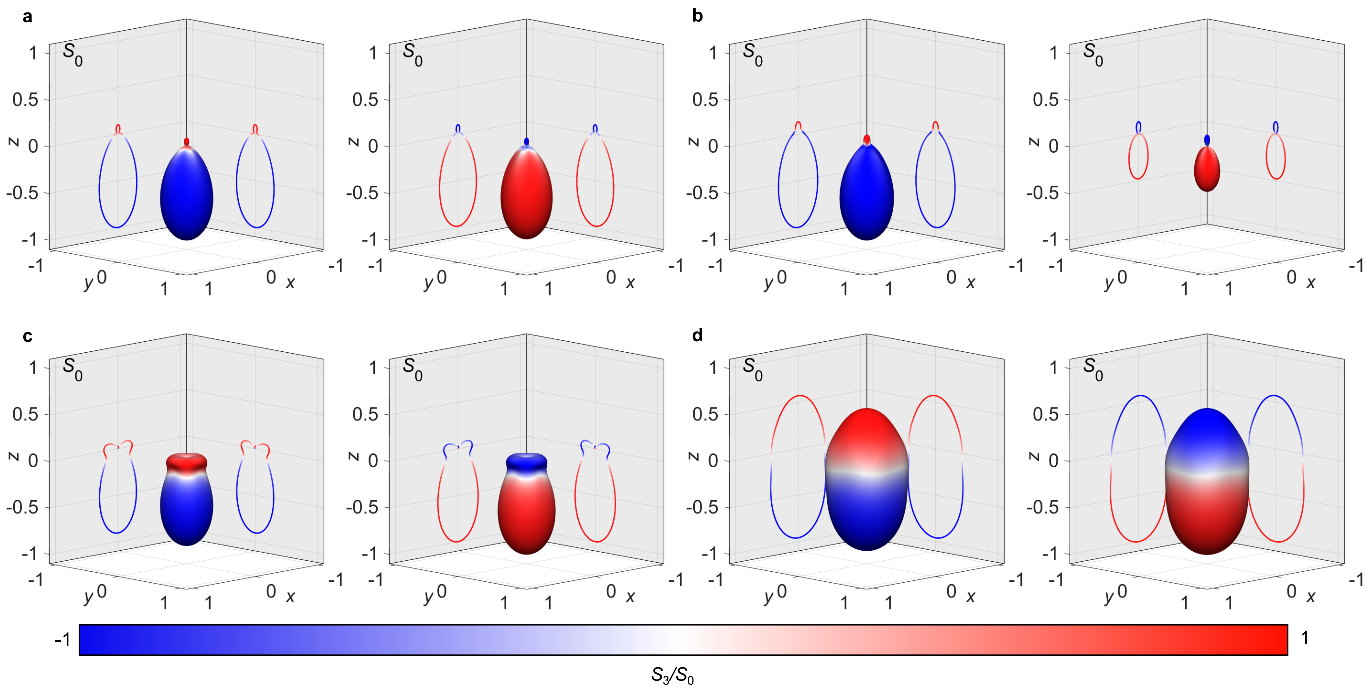

Supplementary Fig. 11. **Spectral dependence of scattering from L-nanohelicoid. a–d** Far-field scattering intensity and polarisation diagrams for a 180 nm L-nanohelicoid in a SiO_2 matrix under (**a**) 500 nm, (**b**) 620 nm, (**c**) 860 nm and (**d**) 950 nm (left column) LCP and (right column) RCP illumination. The colour indicates the polarisation state of the scattered light calculated from Stokes parameters S_3/S_0 (1: RCP; -1: LCP).

a

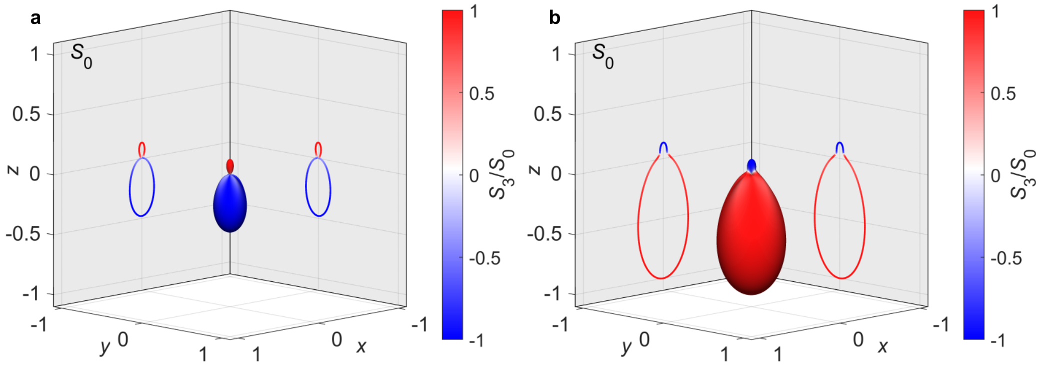

Supplementary Fig. 12. **Numerically simulated scattering on D-nanohelicoid.** Far-field scattering intensity and polarisation diagrams of a D-helicoid calculated under (**a**) LCP and (**b**) RCP 620-nm plane wave illumination. The colour indicates the polarisation state of the scattered light calculated from Stokes parameters S_3/S_0 (1: RCP; -1: LCP).

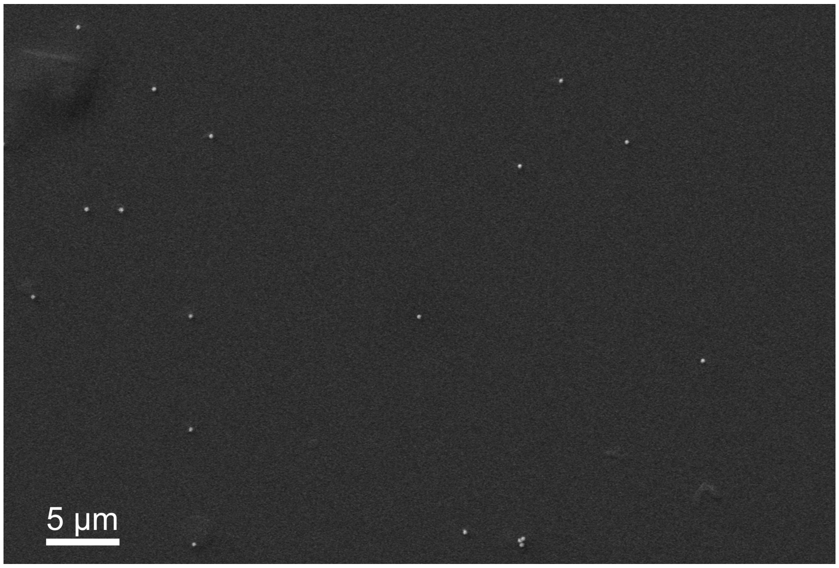

Supplementary Fig. 13. **Separation of nanohelicoids on substrate after spin coating.** An SEM image of nanohelicoids deposited on a substrate by spin coating. The scale bar is 5 μm.

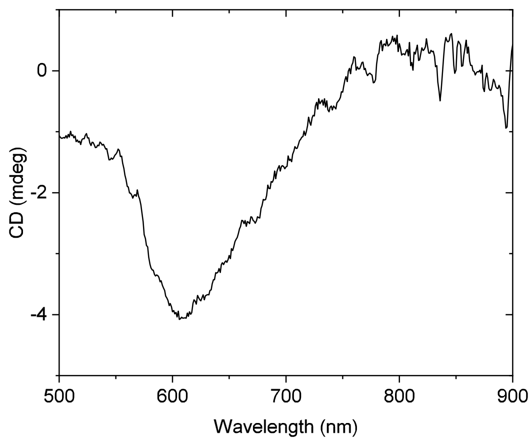

Supplementary Fig. 14. **The experimental CD spectrum of L-nanohelicoid in SiO_2.** The CD spectrum of dispersed L-nanohelicoids on glass with a spin-coated SiO_2 layer on the top. A definition of the handedness of the circularly polarised light from the point of view of the receiver is used, which is a usual definition in CD measurements [10] (which is opposite to the definition used throughout the text). Source data are provided as a Source Data file.

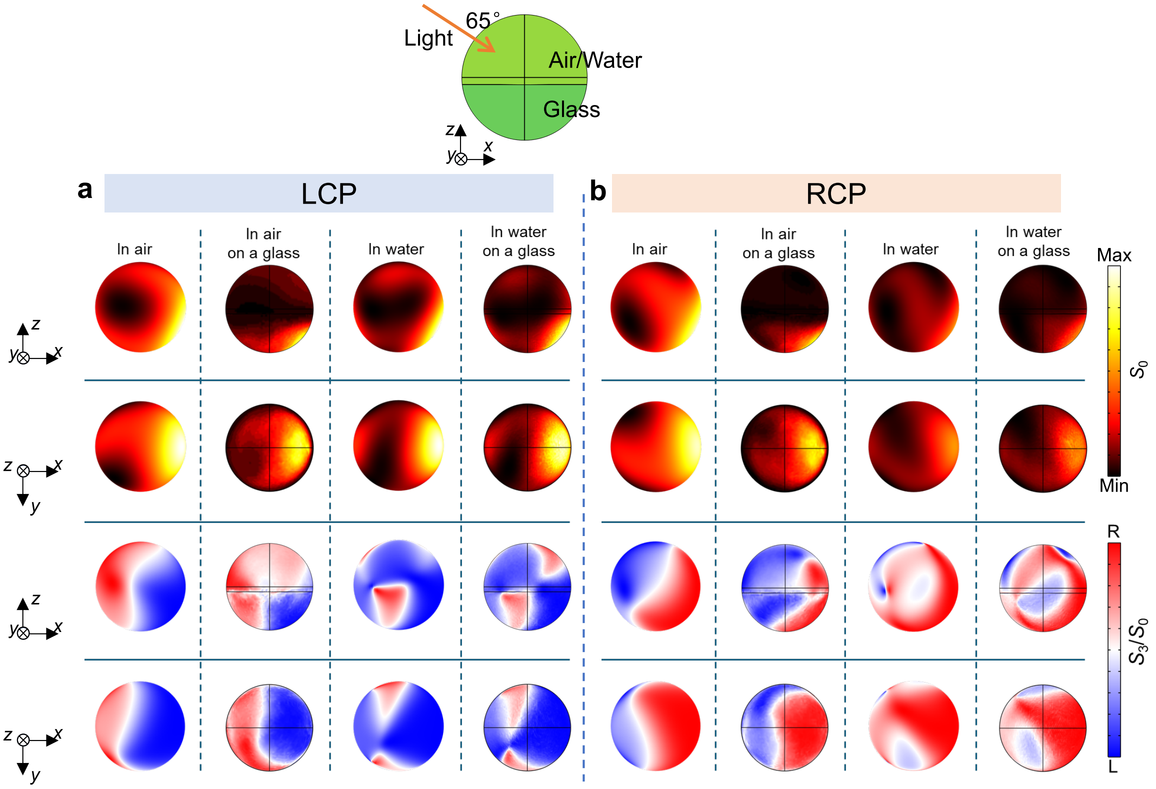

Supplementary Fig. 15. **Influence of the environment and substrate.** Scattering of (a) LCP and (b) RCP light incident at 65° on a nanohelicoid, placed in a uniform matrix or on a substrate made from various media. In the substrate case, the fields were analysed at a distance far enough from the nanohelicoid, so that they are converged to the far-field scattering maps.

Supplementary references

[1] Patoux, A. *et al.* Polarizabilities of complex individual dielectric or plasmonic nanostructures. *Phys. Rev. B* **101** (2020).

[2] Evlyukhin, A. B., Fischer, T., Reinhardt, C. & Chichkov, B. N. Optical theorem and multipole scattering of light by arbitrarily shaped nanoparticles. *Phys. Rev. B* **94** (2016).

[3] Alaee, R., Rockstuhl, C. & Fernandez-Corbaton, I. An electromagnetic multipole expansion beyond the long-wavelength approximation. *Opt. Commun.* **407**, 17–21 (2018).

[4] Zanganeh, E. *et al.* Nonradiating sources for efficient wireless power transfer. *Nanophotonics* **10**, 4399–4408 (2021).

[5] Liu, W. & Kivshar, Y. S. Generalized Kerker effects in nanophotonics and meta-optics. *Opt. Express* **26**, 13085–13105 (2018).

[6] Liu, W. *et al.* Ultra-directional forward scattering by individual core-shell nanoparticles. *Opt. Express* **22**, 16178–16187 (2014).

[7] Wang, D. *et al.* Modified method for computing the optical force of the plasmonics nanoparticle from the maxwell stress tensor. *J. Opt. Soc. Am. B* **34**, 178–182 (2017).

[8] Ye, Q. & Lin, H. On deriving the maxwell stress tensor method for calculating the optical force and torque on an object in harmonic electromagnetic fields. *Eur. J. Phys.* **38**, 045202 (2017).

[9] Nieto-Vesperinas, M. & Xu, X. The complex maxwell stress tensor theorem: the imaginary stress tensor and the reactive strength of orbital momentum. a novel scenery underlying electromagnetic optical forces. *Light Sci. Appl.* **11**, 297 (2022).

[10] Sun, X. *et al.* Tunable reversal of circular dichroism in the seed-mediated growth of bichiral plasmonic nanoparticles. *ACS Nano* **16**, 19174–19186 (2022).
